# Outcomes in dogs undergoing surgical stabilization and non-stereotactic radiation therapy for axial and appendicular bone tumors

**DOI:** 10.3389/fvets.2023.1283728

**Published:** 2024-01-11

**Authors:** Maureen A. Griffin, Andrea Mastorakis, Brandan Wustefeld-Janssens, Tiffany Wormhoudt Martin, Lili Duda, Bernard Seguin, Giovanni Tremolada

**Affiliations:** ^1^Department of Clinical Sciences and Advanced Medicine, University of Pennsylvania School of Veterinary Medicine, Philadelphia, PA, United States; ^2^Department of Clinical Sciences, Flint Animal Cancer Center, Colorado State University, Fort Collins, CO, United States

**Keywords:** bone tumor, canine, pathologic fracture, radiation therapy, surgery

## Abstract

**Background:**

Information on dogs that undergo radiation therapy (RT) with non-stereotactic protocols in addition to surgical stabilization with implant placement for treatment of bone tumors is limited.

**Objective:**

Our primary objectives were to describe the clinical characteristics as well as short- and long-term outcomes, including complications, function, and disease progression, in dogs that underwent both surgical stabilization with implant placement and non-stereotactic RT for local treatment of a bone tumor.

**Methods:**

A bi-institutional retrospective case series was performed.

**Animals:**

Eight client-owned dogs that underwent both surgical stabilization with implant placement and non-stereotactic RT for local treatment of a bone tumor were included.

**Results:**

Tumor types included osteosarcoma or suspected osteosarcoma (5), plasma cell tumor (2), and grade 3 fibrosarcoma (1). Radiation protocols were hypofractionated (palliative intent) in 5 dogs and fractionated (definitive intent) in 3 dogs. Five dogs experienced complications following both RT and surgery, including grade 1 complications in two dogs, a grade 2 complication in one dog, both grade 1 and 2 complications in one dog, and both grade 2 and 3 complications in one dog. Clinical signs subjectively improved in all dogs that had outcomes relative to function documented post-surgery/RT (7). Of these 7 dogs, 4 maintained long-term improvement in function and clinical signs, whereas 3 experienced subsequent recurrence/progression of clinical signs at a median of 133 days (range 91-186) postoperatively in association with biomechanical complications (screw loosening), surgical site infection, and local disease progression in 1 dog each; subsequent treatment resulted in improved clinical signs for each of these 3 dogs, such that overall good long-term functional outcomes were experienced. No dogs required amputation or additional vertebral surgery as salvage for local disease control or palliation. The median progression free interval was 206 days (range 25-1078), and the median survival time was 253 days (range 122-1078) with 1 additional dog lost to follow-up at 575 days. Two dogs experienced local disease progression, and 6 dogs experienced systemic disease progression; both dogs that developed local disease progression received palliative intent RT protocols.

**Clinical relevance:**

In this cohort, dogs with primary bone tumors that underwent surgical stabilization with implant placement and hypofractionated or fractionated non-stereotactic RT for local treatment had a low incidence of major complications, good limb function and ambulation post-treatment, and relatively prolonged survival times despite disease progression.

## Introduction

The most common local treatment modalities for bone tumors in dogs involve surgery or radiation therapy (RT). Owner decision-making regarding which local therapy to pursue is based on a combination of factors including diagnosis, disease stage, prognosis, comorbidities (particularly orthopedic/neurologic that may affect functional outcome with amputation), owner goals, and financial considerations. Limb salvage treatment options include RT and surgical limb salvage, particularly for distal radial and distal extremity tumors as well as tumors involving structures that are not required for weight bearing (1–7). In the setting of pathologic fracture, surgical stabilization alone does not address the local neoplastic disease, leading to persistent pain and lameness or limb disuse in the majority of dogs and poor long-term outcomes (8). Radiation alone is also not considered a viable option for local treatment in the setting of pathologic fracture, as it does not address the instability and associated discomfort. Limb-sparing surgery, involving tumor resection and reconstruction of the bony column, can be considered for dogs with pathologic fracture, and one study reported that only 1/4 dogs with preoperative pathologic fractures developed local recurrence following limb-sparing surgery with metal endoprostheses (5). However, limb-sparing surgery with tumor excision and implant placement is predominantly feasible for distal radial bone tumors and carries a high risk of surgical site infection (up to 78%) and implant-related complications (up to 41%) (5). Non-distal radial tumor locations, the relatively high rate of complications, and substantial cost associated with surgery and postoperative management preclude many dogs from receiving this limb salvaging treatment option.

To date, several studies have demonstrated poor outcomes with a high incidence of major complications following stereotactic RT, or stereotactic body RT (SBRT) when performed for tumors in locations other than the head, in conjunction with surgical stabilization of bone tumors (9, 10). One study on dogs with appendicular osteosarcoma reported deep infection in 5/6 dogs and implant failure in 3/6 dogs following pathologic fracture repair post-SBRT (9). An additional study on dogs with primary appendicular bone tumors treated with SBRT and surgical stabilization reported major complications in 15/17 dogs (10). Due to the high complication rate, treatment of appendicular bone tumors with SBRT and surgical stabilization is not recommended (10). Another study on 16 dogs undergoing pathologic fracture repair included 5 dogs that underwent concomitant RT (8). In that study, the specific radiation protocols and timing of RT relative to surgery were not described, and outcomes relative to limb function and complications were not reported. (8).

For dogs with vertebral tumors, combined surgical and radiation treatment has also been documented, though no reports have clearly described outcomes of dogs undergoing vertebral stabilization with implant placement in addition to RT. In a study on 20 dogs with vertebral tumors, surgery was performed in 12 dogs and RT was performed in 16 dogs (11). Stabilization was performed as a component of surgery in only 3 dogs, and whether these dogs also received RT was not described (11). An additional study on palliative surgical decompression for 22 dogs with primary vertebral osteosarcoma included 7 dogs treated with both surgery and RT (12). However, only 4 dogs in this study had surgical stabilization with implant placement, and it was not documented whether these dogs also received RT (12).

Overall, information is lacking for dogs undergoing surgical stabilization with implant placement in addition to RT using non-stereotactic (either hypofractionated or fractionated) protocols for local treatment of bone tumors. Because of the important differences in radiation dosing and delivery to normal tissues and the subsequent biologic effects, non-stereotactic RT modalities should not be assumed equivalent to stereotactic RT modalities relative to risk of adverse events and outcomes. Our primary objectives were to describe the clinical characteristics as well as short- and long-term outcomes in dogs that underwent both surgical stabilization with implant placement and non-stereotactic RT techniques for local treatment of an appendicular or axial bone tumor. We aimed to evaluate adverse events and functional outcomes associated with this treatment.

## Methods

The medical record database of the Colorado State University James L. Voss Veterinary Teaching Hospital and University of Pennsylvania Ryan Veterinary Hospital were retrospectively searched to identify dogs that underwent surgical stabilization and non-stereotactic RT (either palliative or definitive intent) for treatment of an axial or appendicular bone tumor. All dogs that had both treatment modalities in any order performed and had post-treatment follow-up information were included in the study. Dogs that received SBRT and surgical stabilization were excluded. Information obtained from the medical records included signalment, history of orthopedic or neurologic disease, type and duration of clinical signs, physical examination findings, preoperative diagnostic results, surgical procedures and RT protocols performed, timing of and indication for surgical procedures and RT, cytologic and histopathologic results, neoadjuvant and adjuvant oncologic treatments, complications, progression of local and distant disease, post-treatment limb function and gait, and timing and cause of death or loss to follow-up. In cases with adequate information, lameness was subjectively graded on a 0–5 scale (Supplementary Table 1); when this information was lacking, the lameness was described (13). Neurologic deficits were described. Radiation protocols were defined as palliative intent if they were hypofractionated and administered in 6–10 Gy per fraction for 1–6 total fractions. Radiation protocols were defined as definitive intent if they were fractionated and administered in 2–5 Gy per fraction for at least 10 total fractions. Complications were listed as grades 1–3 in accordance with the Veterinary Radiation Therapy Oncology Group (VRTOG) criteria for acute (within 90 days of RT) and late (more than 90 days following RT) radiation-associated complications, and as grades 1–4 in accordance with the CLASSIC (Classification for Intraoperative Complications) criteria for intraoperative complications and the Accordion criteria for postoperative complications (14, 15).

Progression free interval was defined as days from surgery or RT completion (whichever occurred second) to local or systemic disease progression. Survival time was defined as days from surgery or RT completion (whichever occurred second) to death or euthanasia. Follow-up time was defined as days from surgery or RT completion (whichever occurred second) to last follow-up in dogs that were alive at last follow-up.

Descriptive statistics were calculated for all measured variables. Continuous variables were reported as median (range) and categorical variables were reported as number with or without percentage.

## Results

Eight dogs met inclusion criteria. Dog breeds included Labrador retriever (2), mixed breed (2), miniature schnauzer (1), spinone Italiano (1), saint bernard (1), and mastiff (1). Dogs were female spayed (4), male castrated (3), and female intact (1). At the time of presentation to the referral hospital, the median age was 8.0 years (range 3.3–13.5). Orthopedic/neurologic comorbidities included osteoarthritis in 3 dogs and marked pelvic limb lameness and deformity following trauma in 1 dog. All dogs were presented to the referral hospital for clinical signs, including lameness in 6 dogs, paresis/ataxia in 2 dogs, and a swelling or mass effect in 2 dogs. Median duration of clinical signs prior to presentation to the referral hospital was 35 days (range 15–126).

On physical examination at the referral hospital, the median body weight was 30.1 kg (range 7.4–58.0). One dog was hyperthermic (103.4°F), and vital parameters were within normal limits for all other dogs. Lameness score was recorded in 4 dogs with a median grade of 2.5 (range 2–3). A swelling or mass effect associated with a bone tumor was noted in 2 dogs (both with distal radial lesions). One dog appeared uncomfortable and reluctant to sit (this dog had a lumbar vertebral lesion), and one dog had delayed proprioception in all limbs with marked ataxia and apparent neck pain with reluctance to move the neck (this dog had a cervical vertebral lesion). No additional neurologic abnormalities were noted. One dog (with a proximal humeral lesion) had a palpably enlarged superficial cervical lymph node. No other significant systemic findings were noted on physical examination.

The primary tumor was diagnosed as osteosarcoma in 3 dogs, plasma cell tumor in 2 dogs, suspected osteosarcoma on the basis of clinical and imaging characteristics without cellular diagnosis in 2 dogs, and grade 3 fibrosarcoma/soft tissue sarcoma in 1 dog. Cellular diagnosis was obtained via histopathology in all 6 dogs with diagnoses. Histopathology was obtained via preoperative incisional biopsy in 3 dogs, intraoperative incisional biopsy in 2 dogs, and both preoperative and intraoperative incisional biopsy in 1 dog. No dogs had excisional biopsies performed, though 1 dog with an axial tumor underwent substantial cytoreduction to relieve spinal cord compression. Primary tumor location was distal radius in 2 dogs (osteosarcoma and suspected osteosarcoma without diagnosis), proximal humerus in 2 dogs (osteosarcoma and suspected osteosarcoma without diagnosis), vertebrae in 2 dogs (L5 plasma cell tumor and C2-3 fibrosarcoma), proximal radius in 1 dog (plasma cell tumor), and distal femur in 1 dog (osteosarcoma).

Imaging modalities used to characterize the bone lesion prior to treatment included radiographs (6) and CT (3). For both dogs with vertebral lesions, an aggressive osseous lesion with secondary spinal cord deviation and compression was noted on CT (2) and MRI (1). For dogs with appendicular lesions, an aggressive bone lesion with pathologic fracture was noted in 3 dogs, an aggressive bone lesion without pathologic fracture was noted in 1 dog, and fracture without an overtly aggressive etiology was noted in 2 dogs (neither of these dogs had images that were reviewed by a radiologist prior to local treatment). No dogs had overt evidence of metastatic or multifocal disease on staging prior to initial treatment, though one dog did not have thoracic imaging performed prior to local treatment. For the 2 dogs with plasma cell tumors, systemic assessment prior to local treatment included complete blood count, biochemistry panel, urinalysis, Bence-Jones protein testing, serum protein electrophoresis, bone marrow cytology, thoracic radiographs, lumbar vertebral radiographs, joint fluid analysis (1 dog), and lymph node cytology (1 dog); diagnostic results were not consistent with multiple myeloma in either dog. For these 2 dogs, neoadjuvant prednisone and melphalan were started prior to and continued after local treatment. For 1 dog, prednisone (0.7 mg/kg/day PO) and melphalan (0.3 mg/kg PO every other day for 10 days followed by every 4 days) were both initiated within 17 days of surgical stabilization (which was performed 28 days prior to RT); these medications were continued long-term. For the other dog, prednisone (0.4 mg/kg/day PO) and melphalan (0.4 mg/kg PO daily for 5 days every 21 days) were both initiated within 6 days of RT (which was completed 274 days prior to surgical stabilization); for this dog, prednisone and melphalan were discontinued within approximately 180 days of initial treatment. No neoadjuvant tumor-directed treatments were administered for the remaining dogs.

All dogs underwent local surgery with implant placement and RT. Five dogs underwent surgery followed by RT, with a median time between surgery and subsequent RT initiation of 26 days (range 19–196). The other 3 dogs underwent RT followed by surgery, with a median time between initial RT completion and subsequent surgery of 274 days (range 0–500). Four dogs were treated with planned surgery in combination with RT, 2 dogs were initially treated with RT alone and later underwent surgery for pathologic fracture repair (1 of these dogs also underwent repeated RT), and 2 dogs were initially treated with surgery alone for stabilization of fractures not known to be pathologic and subsequently received RT for treatment of the bone tumors. Radiation protocol intent was palliative in 5 and definitive in 3, and RT data is provided in Supplementary Data Sheet 1.

The 4 dogs that were treated with planned surgery and RT included both dogs with vertebral tumors and 2 dogs with appendicular osteosarcoma. For the 2 dogs with vertebral tumors, surgery was performed first and involved tumor cytoreduction and hemilaminectomy for spinal cord decompression in addition to vertebral stabilization. For the dog with the L5 plasma cell tumor, stabilization was performed with 2 screws in both the L4 and L6 vertebral bodies, k-wires placed across the screws and maintained in position with cerclage wires, and polymethyl methacrylate applied over the pin/screw scaffold. For the dog with the C2-3 fibrosarcoma, stabilization was performed with 8 positive profile transfixation pedicle pins from C2-6, 2 smooth medial-lateral pins, 2 smooth parasagittal pins, and polymethyl methacrylate mixed with cefazolin. For these two dogs, definitive intent RT protocols were subsequently started 28 days and 19 days postoperatively, respectively. The dog with the L5 tumor received fractionated RT with a dose of 3 Gy for 12 fractions (total dose 36 Gy) administered every other day (Monday–Wednesday–Friday), and the dog with the C2-3 tumor received intensity-modulated RT (IMRT) with a dose of 2.8 Gy for 20 fractions (total dose 56 Gy) administered daily (Monday through Friday). Another dog that underwent planned surgery and RT had a proximal humeral osteosarcoma and underwent pathologic fracture repair with placement of an intramedullary pin, 2 bone plates, and multiple screws, followed by palliative intent RT beginning 20 days postoperatively with a dose of 8 Gy for 2 fractions on consecutive days (total dose 16 Gy). The final dog that underwent planned surgery and RT had a distal radial osteosarcoma and underwent palliative intent IMRT with a prescription of 8 Gy for 2 fractions on consecutive days (total dose 16 Gy) and pathologic fracture fixation under the same anesthetic event as the second RT fraction. In this surgery, a broad limb salvage plate was bent and fixed to the cranial radius and dorsal third metacarpal bone with multiple locking screws to create an arthrodesis.

Of the 2 dogs that underwent RT alone initially followed by surgical pathologic fracture repair, one had a proximal radial plasma cell tumor. Radiographs revealed an aggressive bone lesion characterized predominantly by lysis extending to the level of the radial head; a large segment of bone was not present caudally, and circumferential cortical thinning with a cranial cortical discontinuity was noted. This dog underwent definitive intent RT with a dose of 4 Gy for 11 fractions (total dose 44 Gy) administered every other day (Monday–Wednesday–Friday). This dog’s lameness improved initially but subsequently worsened approximately 101 days following treatment. Supportive care was elected for several additional months, until radiographs obtained 235 days following treatment revealed pathologic fracture without overt disease recurrence/progression. A splint and bandages were maintained for 38 days, and pathologic fracture repair was performed 274 days following RT. Fracture repair involved cancellous bone graft instillation into a 1.5 cm bone defect and fixation of a complete, simple, transverse fracture with a broad locking compression plate with multiple cortical and locking screws. The additional dog that underwent RT alone initially followed by surgical pathologic fracture repair had a distal radial suspected osteosarcoma (no cellular diagnosis was obtained) without evidence of pathologic fracture on limb radiographs. This dog underwent palliative intent RT with a dose of 8 Gy for 2 fractions on consecutive days (total dose 16 Gy). The dog’s lameness improved following RT, but acute apparent pain, increased tumor size, and progressive lameness were noted 499 days following RT. Radiographs revealed an aggressive lesion with significant cortical lysis and an intra-articular pathologic fracture. The dog subsequently underwent another course of palliative intent RT with a dose of 10 Gy in a single fraction, and the following day (500 days after initial RT completion) the dog underwent surgical stabilization via arthrodesis with a hybrid carpal arthrodesis plate applied to the radius, radiocarpal bone, and third metacarpal bone with cortical screws.

Of the two dogs that underwent surgery for presumed non-pathologic fractures with subsequent RT following diagnosis of neoplasia, both had radiographs performed by the primary veterinarian and not evaluated by a radiologist, and both underwent surgical treatment prior to presentation to the referral hospital. One had a proximal humeral, complete, long oblique, diaphyseal fracture, and the other had a distal femoral, complete, diaphyseal/metaphyseal fracture. The dog with the proximal humeral fracture underwent repair with an intramedullary pin placed in retrograde fashion and four cerclage wires. This dog had a mild lameness 59 days postoperatively and recheck radiographs revealed possibly wider fracture line suspected to be associated with motion of the fragments. The dog continued to have a mild lameness, and 105 days postoperatively radiographs reportedly revealed a healed fracture (no radiologist report was obtained). The dog’s activity was then increased, and 168 days postoperatively grade 3–4 lameness was noted. Radiographs at this time revealed an aggressive lesion of the proximal humeral metaphysis at the proximal extent of the prior fracture site (no radiologist report was obtained). Radiographic appearance was noted to be consistent with osteosarcoma, though no cellular diagnosis was performed. The dog was subsequently presented to the referral hospital, and palliative intent RT was initiated 196 days postoperatively with a dose of 8 Gy for 2 fractions on consecutive days (total dose 16 Gy). The dog with the femoral fracture underwent repair with 3 cerclage wires and a distal femoral bone plate applied with cortical screws. Intraoperatively, soft bone was noted at the distal femoral metaphysis and a sample was submitted for histopathology, which was consistent with osteosarcoma. Thoracic staging was not performed in this dog prior to surgery. The dog was presented to the referral hospital 25 days postoperatively (following neoplastic diagnosis) and had a grade 3 lameness at the time of presentation. Thoracic radiographs were obtained at this time and revealed pulmonary metastatic disease. The dog subsequently underwent palliative intent RT with a dose of 8 Gy for 2 fractions on consecutive days (total dose 16 Gy).

All dogs survived to discharge following surgery and RT. The median postoperative hospitalization time was 1 day (range 1–5). Overall, 5 dogs experienced complications associated with surgery (and not attributed to anesthesia): 1 dog experienced intraoperative complications and 4 dogs experienced postoperative complications. However, 2 of these dogs underwent surgery followed by RT, such that the surgical complications could not be attributed to neoadjuvant RT for these dogs (Of these 2 dogs, one experienced two grade 2 intraoperative complications [hemorrhage and fracture of a vertebral facet], and one experienced both a grade 1 complication within 30 days of surgery [large seroma] and a grade 2 complication more than 30 days postoperatively [subtle lameness with widening of the fracture gap]). Therefore, only 3 dogs experienced surgical complications after RT. In these 3 dogs, complications occurred postoperatively within 30 days of surgery in all 3 dogs, and complications occurred more than 30 days after surgery in 2 dogs. Postoperative complications that occurred after hospitalization within 30 days of surgery included one grade 1 complication (minimal dehiscence of the skin incision) and two grade 2 complications (surgical site infection and discharge from the surgical site). For the 2 dogs that developed postoperative complications more than 30 days after surgery and following RT, one had a grade 2 complication (surgical site infection first documented 91 days postoperatively in a dog that underwent palliative intent RT [8 Gy x 2 fractions] and concurrent surgical stabilization) and one had a grade 3 complication (screw loosening requiring subsequent surgical removal 280 days postoperatively in a dog that underwent definitive intent RT [4 Gy x 11 fractions] and surgery 274 days after RT completion).

Three dogs, all of which received definitive intent RT protocols, experienced acute complications associated with RT: all were grade 1 complications related to skin toxicity. Two of these acute grade 1 skin adverse events occurred following both surgery and RT, whereas one occurred prior to surgical stabilization. For the dog that developed a pathologic fracture following definitive intent RT of its proximal radial plasma cell tumor, it is unknown if this fracture occurred due to bone necrosis secondary to RT (such that it would be classified as a grade 3 late RT complication) or due to the underlying neoplastic etiology with bone lysis. However, this fracture occurred after RT alone and prior to surgical stabilization. No additional dogs experienced late RT complications.

In summary, 5 dogs experienced surgical and/or RT-associated complications after both surgical and radiation treatments were performed. These included grade 1 VRTOG complications in two dogs, a grade 2 Accordion complication in one dog, grade 1 and 2 Accordion complications in one dog, and grade 2 and 3 Accordion complications in one dog. Both dogs that experienced grade 1 adverse events alone had acute grade 1 VRTOG skin changes following definitive intent RT protocols that were initiated 19 and 28 days postoperatively. The dog that experienced a grade 2 Accordion adverse event within 30 days of surgery (surgical site discharge) underwent surgery 1 day after palliative intent RT, which was performed 499 days following an initial course of palliative intent RT. The dog that experienced a grade 1 Accordion adverse event within 30 days of surgery (slight incisional dehiscence) and grade 2 Accordion adverse event more than 30 days after surgery (surgical site infection) underwent surgery on the second and final day of a palliative intent RT protocol. The dog that experienced a grade 2 Accordion adverse event within 30 days of surgery (surgical site infection) and grade 3 Accordion adverse event more than 30 days after surgery (screw loosening) underwent pathologic fracture stabilization 274 days following definitive intent RT completion.

Two dogs received additional courses of RT. One dog received an additional course of definitive intent RT (3.5 Gy/fraction for 12 fractions [total dose 42 Gy] administered every other day [Monday–Wednesday–Friday]) for a second plasma cell tumor lesion that developed on the dog’s ischium approximately 292 days following completion of definitive intent RT for its L5 plasma cell tumor. One dog received an additional course of RT for the same lesion that was previously treated with RT and surgery. This dog had a distal radial tumor (suspected osteosarcoma with no cellular diagnosis) that was treated with palliative intent RT followed by another course of palliative intent RT and surgery 499 days later. Approximately 186 days later, the dog developed a grade 1 lameness with a firm and painful swelling of the distal radius, consistent with disease progression, and an additional RT fraction (10 Gy) was administered for palliation, with no subsequent complications documented.

Adjuvant treatments were performed in 5 dogs, including chemotherapy and bisphosphonates in 2 dogs, chemotherapy alone in 2 dogs, and HER2/neu recombinant Listeria vaccine in 1 dog with osteosarcoma. The 2 dogs that received neoadjuvant prednisone and melphalan for treatment of plasma cell tumors continued to receive these medications post-treatment and also received pamidronate following local therapy (3 doses in 1 dog, 4 doses in the other dog). The dog with a plasma cell tumor that received an additional course of RT for a new lesion also received doxorubicin (6 doses total) starting at the time of RT for its second bone lesion. The dog with the C2-3 grade 3 fibrosarcoma received a single dose of doxorubicin at the time of documented pulmonary metastatic disease 24 days following definitive intent RT completion. The dog with distal femoral suspected osteosarcoma (with no cellular diagnosis) that had surgery performed to repair a fracture not known to be pathologic followed by palliative intent RT and diagnosis of pulmonary metastatic disease received metronomic cyclophosphamide for greater than 180 days. One dog with proximal humeral osteosarcoma was enrolled in a HER2/neu Listeria vaccine clinical trial and received 8 vaccinations over the course of 148 days; no additional adjuvant treatments were performed. No significant complications were documented in association with any of these adjuvant treatments.

Following RT and surgery, all dogs had subjectively adequate ambulation and use of the limb(s). Seven dogs had outcomes relative to function documented long-term postoperatively; for the dog with the proximal humeral lesion (suspected osteosarcoma) that underwent surgical repair for a fracture that was not known to be pathologic in nature and subsequent palliative intent RT, follow-up regarding limb use and gait is lacking between RT and euthanasia 130 days post-RT. Clinical signs improved in all 7 dogs that had long-term functional outcomes documented post-surgery/RT. Significant improvement in lameness and/or ambulation was reported without recurrence or progression of clinical signs associated with the bone lesion that was treated with surgery and RT in 4 dogs. In 3 dogs, clinical signs recurred and progressed after an initial period of clinical improvement at a median of 133 days (range 91–186) following surgery. This was associated with biomechanical complications (screw loosening) in 1 dog 133 days following treatment, and lameness progressed until the screw was subsequently removed 279 days postoperatively; following this procedure, no concerns were noted in association with the bone tumor site. In another dog, progression of clinical signs was associated with a surgical site infection that was documented 91 days postoperatively, and clinical signs improved with long-term antimicrobial administration; the dog was noted to be ambulating well with no apparent pain 575 days post-treatment. The additional dog that developed recurrence and progression of clinical signs following surgery and RT was the dog that received a palliative intent course of RT for its distal radial tumor, subsequent palliative intent RT and surgery for a pathologic fracture 499 days following initial RT, and then developed disease progression 186 days post-surgery, at which time it received a third course of palliative intent RT. No dogs (aside from the dog that had a screw removed) underwent subsequent surgical procedures or amputation for local disease treatment or palliation.

Disease progression was documented in 7 dogs following RT and surgery, and the median progression free interval was 206 days (range 25–1,078). Local disease progression was reported in 2 dogs at 186 and 378 days. These dogs both underwent palliative intent RT in combination with surgery. Systemic or metastatic disease progression was reported in 6 dogs at a median of 218 days (range 25–1,078). Both local and systemic disease progression was documented in 1 dog. Both dogs with plasma cell tumor lesions developed evidence of multiple myeloma, 1 dog with proximal humeral osteosarcoma developed multiorgan metastatic disease, and 3 dogs (one with distal radial suspected osteosarcoma, one with distal femoral osteosarcoma, and one with C2-3 grade 3 fibrosarcoma) developed new onset or progression of pulmonary metastatic disease following local treatment. At study completion, 7 dogs were dead (all euthanized) with a median survival time of 253 days (range 122–1,078) and 1 dog was still alive with last follow-up 575 days post-treatment. Of the 3 dogs (including 2 with plasma cell tumors and 1 with fibrosarcoma) that received definitive intent RT, the median survival time was 883 days (range 122–1,078). Of the 5 dogs (including 3 with osteosarcoma and 2 without definitive diagnosis) that received palliative intent RT, the median survival/follow-up time was 253 days (range 130–575). Complication, disease progression, and survival/follow-up data has been provided for each dog relative to treatment performed and tumor type and location (Table 1).

**Table 1:** Data for each dog including tumor type and location, surgery and RT descriptions and dates of treatment, reported complications, neoadjuvant and adjuvant therapies, time to disease progression and type of disease progression, and survival/follow-up data.

Dog Number	Tumor type and location	Surgery description and date	RT protocol intent and dates	Complications reported relative to surgery and RT. *Complications following both surgery and RT	Neoadjuvant/ adjuvant therapy	Time to disease progression (days) and local (L) vs. systemic (S)	Survival (S) or follow-up (F) time (days)
1	Plasma cell tumor, L5	7/17/07: Spinal stabilization L4-6, hemilaminectomy	8/13/07–9/7/07: definitive RT (3 Gy x 12)	*Acute RT Grade 1.	Melphalan, prednisone, pamidronate, doxorubicin	292 (S)	883 (S)
2	Plasma cell tumor, left proximal radius	8/29/08: Fracture stabilization with bone plate/screws, cancellous graft	11/5/07–11/30/07: definitive RT (4 Gy x 11)	Acute RT Grade 1. *Postoperative Grade 2 (< 30 days), Grade 3 (> 30 days).	Melphalan, prednisone, pamidronate	1,078 (S)	1,078 (S)
3	No cellular diagnosis, right distal radius	1/29/10: Fracture stabilization and arthrodesis with hybrid transcarpal bone plate/screws	9/16/08–9/17/08: palliative RT (8 Gy x 2) 1/28/10: palliative RT (10 Gy x 1)	*Postoperative Grade 2 (< 30 days).	None	186 (L), 229 (S)	229 (S)
4	Grade 3 fibrosarcoma, C2-3	10/21/11: Spinal stabilization C2-6, hemilaminectomy	11/8/11–12/6/11: definitive as IMRT (2.8 Gy x 20)	Intraoperative Grade 2. *Acute RT Grade 1.	Doxorubicin (1 dose)	25 (S)	122 (S)
5	No cellular diagnosis, left proximal humerus	7/13/12: Fracture stabilization with intramedullary pin and cerclage	1/24/13–1/25/13: palliative RT (8 Gy x 2)	Postoperative Grade 1 (both <30 days and > 30 days).	None	Not reported	130 (S)
6	Osteosarcoma, right distal femur	12/20/13: Fracture stabilization with bone plate/screws, cerclage, and synthetic bone graft	1/14/14–1/15/14: palliative RT (8 Gy x 2)	None	Metronomic cyclophosphamide	122 (S)	497 (S)
7	Osteosarcoma, right proximal humerus	12/25/13: Fracture stabilization with bone plates/screws and intramedullary pin	1/14/14–1/15/14: palliative RT (8 Gy x 2)	None	HER2/neu Listeria vaccine clinical trial	206 (S)	253 (S)
8	Osteosarcoma, right distal radius	6/3/21: Fracture stabilization and arthrodesis with limb salvage bone plate/screws	6/2/21–6/3/21: palliative as IMRT (8 Gy x 2)	*Postoperative Grade 1 (< 30 days), Grade 2 (> 30 days).	None	378 (L)	575 (F)

## Discussion

In this cohort, dogs with primary bone tumors that underwent surgical stabilization with implant placement in addition to non-stereotactic RT for treatment of their local disease overall had good outcomes with a low incidence of major complications, subjectively good limb function and ambulation post-treatment, and relatively prolonged survival times despite progressive disease. Local disease control was relatively robust in this population of dogs. The only dogs that experienced documented local disease progression underwent palliative intent, rather than definitive intent, RT protocols. Local disease progression is an anticipated outcome with this modality of RT in which the goal is to provide local palliation and improved quality of life rather than long-term local disease control. All other dogs experienced disease progression associated with systemic disease, and adjuvant chemotherapy was performed in only 4 dogs with varying protocols (including 1 dog that received chemotherapy following documentation of metastatic disease). The overall median survival and follow-up times were relatively prolonged, with multiple dogs experiencing long-term outcomes despite disease progression.

Functional outcomes following surgical stabilization and RT were overall very good. All dogs experienced initial improvement in clinical signs. Of the 7 dogs with long-term functional outcomes reported, 4 maintained improvement in function and clinical signs, whereas 3 experienced subsequent recurrence/progression of clinical signs at 91–186 days postoperatively in association with postoperative complications (1 due to surgical site infection and 1 due to biomechanical failure with screw loosening) or local disease progression. Subsequent treatment of the complication or disease progression resulted in improvement in clinical signs, such that overall good long-term functional outcomes were experienced by these dogs. No dogs required amputation or additional vertebral surgery as a salvage treatment for local disease control or palliation.

No intraoperative complications occurred in dogs that had previously received RT. Complications occurred following both surgery and RT in 5 dogs, but the majority of complications were grade 1 (n = 3) and grade 2 (n = 3), and only one grade 3 complication occurred (screw loosening requiring removal). Two dogs developed surgical site infections and an additional dog had mild discharge from the incision post-treatment; one of the dogs with surgical site infection also developed screw loosening. Aside from the dog that underwent screw removal, these dogs were managed with systemic antimicrobials and supportive care, and they continued to have good function of their limbs without requirement for additional local intervention. Ultimately, no dogs developed major complications following surgery and RT that resulted in an indication for radical surgical procedures (such as amputation) or death/euthanasia.

Based on the results of this cohort, surgical stabilization in conjunction with non-stereotactic RT protocols (both hypofractionated and fractionated) appears to result in acceptable complication rates, with a low incidence of major complications, and adequate functional outcomes. This is in stark contrast to the findings of surgical stabilization in conjunction with SBRT, in which major complication rates are prohibitively high. The differences in these RT protocols and their effects on tumor and surrounding normal tissues are presumed responsible for these opposing findings (16–20). It is particularly important to distinguish differences between non-stereotactic protocols relative to stereotactic ablative RT (21). With regards to non-stereotactic RT, responses of tumor and normal tissues have been characterized by the “5 R’s” of radiotherapy: repair of DNA damage, redistribution of cells in the cell cycle, reoxygenation of tumor cells, repopulation of tumor and normal tissues, and intrinsic radiosensitivity of tissues and tumor cells (17, 22, 23). Alternatively, stereotactic RT overcomes radiobiologic limitations via stereotactically verified patient positioning and radiation delivery techniques that result in a minimal volume of normal tissue in the high dose region (17). Therefore, the risks for, and mechanisms of, toxicity have potential to be substantially different for these different radiation modalities.

For non-stereotactic, definitive intent RT protocols, the goal of durable local disease control and improved survival is achieved via fractionation with small doses (< 5 Gy) per fraction, a large number of fractions (≥ 10), and a higher total dose of radiation relative to palliative hypofractionated protocols. In doing so, a high total dose of radiation is delivered to the tumor for disease control, but the normal tissues surrounding the tumor have enhanced ability to repair with the small doses per fraction, thereby reducing toxicity (17). Alternatively, for non-stereotactic, palliative intent RT protocols, the goal of improvement in quality of life and discomfort associated with the local disease without attaining durable local disease control or improved survival is achieved via hypofractionation with moderate (< 10 Gy) doses per fraction, a small number of fractions (< 10), and lower total dose of radiation relative to definitive fractionated protocols. With this type of hypofractionated treatment, the risk of acute side effects is reduced. Late side effects are possible with the higher dose per fraction relative to fractionated protocols, but in many scenarios, given the palliative nature of treatment, life expectancy is not long enough to develop such late RT effects. In contrast to these non-stereotactic fractionated and hypofractionated protocols, stereotactic RT aims to ablate the tumor by administering large doses (≥ 6 Gy) per fraction in a small number of fractions (≤ 5) with short interfraction interval. Given the high dose per fraction, image guidance techniques to deliver radiation within submillimeter accuracy are required. Thus, SBRT protocols aim to reduce side effects by minimizing treatment of normal tissues (i.e., dose avoidance with steep dose drop-off) and targeting the gross tumor as much as possible, rather than via administration of smaller doses per fraction as with finely fractionated RT (17). In addition, IMRT, an advanced form of 3D conformal RT, is a radiation planning technique that involves inverse treatment planning to enhance sculpting of the radiation dose and thereby minimize dose to adjacent normal structures (17). The dose frequency and total dose protocols of IMRT are varied and can be either definitive or palliative intent with fractionation or hypofractionation, respectively.

Although RT-associated adverse effects are possible with each radiation modality, the means of limiting normal tissue radiation dose is substantially different across techniques, and these differences are important in considering the potential for adverse effects of normal tissues that do receive radiation dose. Although the risk of side effects can be reduced with careful conformal planning for SBRT cases, the risk of late effects to normal tissues that do receive radiation is potentially greater with SBRT compared to non-stereotactic RT, owing to the high dose per fraction administered with limited interval between fractions to allow for normal tissue repair (18). Another consideration involves endothelial cell apoptosis via the ceramide signaling pathway that occurs with high irradiation doses (≥ 10 Gy/fraction) delivered via SBRT (24, 25). Though this effect contributes to tumor necrosis, vascular damage also results in microvessel collapse, chronic inflammation, and subsequent ischemia, necrosis, and fibrosis of surrounding normal tissues (26). This effect may contribute to the high rates of major complications for dogs treated with SBRT and surgical stabilization, as endothelial cell apoptosis and the subsequent changes are likely to complicate wound healing and result in substantial morbidity following surgery. An additional consideration is similar to the reasoning for use of non-stereotactic RT protocols in a neoadjuvant setting for non-osseous tumor types (27). Although both stereotactic and fractionated protocols have the potential to administer a large total dose of radiation, fractionated RT allows for greater repair of normal tissues than SBRT, owing to the lower dose per fraction administered with fractionated RT. The ability for normal tissue repair may be important in reducing the risk of complications, such as infection and biomechanical compromise, following surgical stabilization and fractionated RT as compared to SBRT. Finally, hypofractionated, palliative intent (non-stereotactic) protocols are likely well tolerated in conjunction with surgical stabilization owing to the lower total dose of radiation received by normal tissues with a resultant reduced risk for acute adverse effects; though late adverse effects are possible due to the relatively high dose per fraction, survival time is often inadequate (as tumor control is less robust) for dogs to experience these complications.

This study has several limitations. First, due to the retrospective nature, complete clinical information was lacking for some patients. Recheck examinations and diagnostics were not standardized, such that the reported progression free interval is likely to be an overestimation of true progression free interval. In addition, ambulation and gait assessments were based on physical examination findings rather than objective outcome measurements such as force platform gait analysis. Also, due to the small sample size of dogs in this report and the variability in staging, treatments performed, and follow-up, it was not possible to perform statistical analyses with regards to risk factors for complications, disease progression, or survival due to potential for error. Because this cohort varied widely relative to neoplastic disease, management, and follow-up, cause and effect is not clearly determined and prognostic information regarding survival times relative to surgical and/or RT procedures cannot be ascertained. Finally, selection bias may have occurred as all cases were contributed by academic institutions and all owners elected both surgical and radiation treatment modalities.

In conclusion, this report adds clinically important data to the literature on dogs undergoing surgical stabilization in addition to RT for local treatment of bone tumors. Based on the results of this study, surgical stabilization in combination with non-stereotactic RT protocols can be considered for local treatment of bone tumors in dogs. Unlike surgical stabilization in conjunction with SBRT, findings of this cohort suggest that surgery and non-stereotactic (hypofractionated or fractionated) RT does not result in prohibitively high major complication rates, and functional outcomes appear to be acceptable despite the potential for disease progression.

## Data availability statement

The original contributions presented in the study are included in the article/Supplementary material, further inquiries can be directed to the corresponding author.

## Ethics statement

Ethical approval was not required for the studies involving animals in accordance with the local legislation and institutional requirements because all data was obtained in retrospective fashion; no prospective research study was performed. All clients consented to use of their pets’ medical data for research and publication. Written informed consent was obtained from the owners for the participation of their animals in this study.

## Author contributions

MG: Conceptualization, Data curation, Investigation, Methodology, Supervision, Writing – original draft, Writing – review & editing. AM: Data curation, Writing – original draft, Writing – review & editing. BW-J: Conceptualization, Data curation, Investigation, Methodology, Supervision, Writing – original draft, Writing – review & editing. TM: Conceptualization, Data curation, Investigation, Methodology, Supervision, Writing – original draft, Writing – review & editing. LD: Data curation, Supervision, Writing – original draft, Writing – review & editing. BS: Conceptualization, Data curation, Investigation, Methodology, Supervision, Writing – original draft, Writing – review & editing. GT: Conceptualization, Data curation, Investigation, Methodology, Supervision, Writing – original draft, Writing – review & editing.
