# Supplementary material for: Outcomes in dogs undergoing surgical stabilization and non-stereotactic radiation therapy for axial and appendicular bone tumors
Source: Front Vet Sci. 2024 Jan 11;10:1283728. doi: 10.3389/fvets.2023.1283728 (PMC10808726; doi:10.3389/fvets.2023.1283728)
Supplement: Supplementary file 2 [file Data_Sheet_1.DOCX]

Supplementary Material

# Supplementary Tables

Table S1: Lameness scoring^20^

| Grade 0 | No lameness |
| --- | --- |
| Grade 1 | Lameness difficult to observe or inconsistent, off-loading at stance |
| Grade 2 | Lameness consistently observed in some scenarios but not at walk |
| Grade 3 | Lameness consistently observed at trot |
| Grade 4 | Lameness consistently observed at walk, may not weight bear when standing or may place foot |
| Grade 5 | Minimal or non-weight bearing lameness |

Table S2: Radiation therapy data for each dog

[See attached Excel sheet]

GTV: gross tumor volume

CTV: clinical target volume

PTV: planning target volume

ICRU: International Commission on Radiation Units

OAR: organ at risk

PRV: planning organ at risk volume

DVH: dose-volume histogram

SSD: source-skin distance
SAD: source-axis distance
